# Perceived Difficulties in Physical Tasks and Physical Fitness in Treatment- and Non-Treatment-Seeking Youths with Obesity

**DOI:** 10.3390/children9091351

**Published:** 2022-09-04

**Authors:** Giada Ballarin, Maria Rosaria Licenziati, Olivia Di Vincenzo, Luca Scalfi, Giuliana Valerio

**Affiliations:** 1 Department of Movement Sciences and Wellbeing, University of Naples “Parthenope”, 80133 Naples, Italy; 2 Obesity and Endocrine Disease Unit, Department of Neurosciences, Santobono-Pausilipon Children’s Hospital, 80129 Naples, Italy; 3 Department of Clinical Medicine and Surgery, Federico II University of Naples, 80131 Naples, Italy; 4 Department of Public Health, Federico II University of Naples, 80131 Naples, Italy

**Keywords:** obesity, health-related physical fitness, cardiorespiratory fitness, strength, childhood

## Abstract

Youths with obesity are more likely to experience physical and psychosocial distress which strongly limits physical activity, with consequences on the quality of life. Most evidence of lower physical fitness and physical activity levels has been reported in treatment-seeking samples, while few data are available in community samples. Our aim was to assess whether perceived difficulties in physical tasks and physical fitness performance differed between treatment- and non-treatment-seeking youths with obesity, enrolled from a hospital (H) and a school (S). Three hundred fifty-one youths (269 from H and 82 from S) were enrolled. Sports participation, sedentary habits and perceived difficulties in physical tasks were assessed by interview. Six-minute walk test (SMWD) and long jump (LJ) were performed. BMI Z-score, sedentary time and perceived difficulties were higher in H vs. S. In addition, youths from H scored worse in SMWD and LJ. For the same BMI Z-score, the perceived difficulties and physical fitness were poorer in the H compared to the S group. The setting (H) was the stronger predictor of perceived difficulties and lower performance. Our findings underline that physical aspects imposed by obesity are more evident in treatment-seeking youths. Counseling related to perceived difficulties in physical tasks and performance is useful to treat youth with obesity with appropriate and personalized modalities.

## 1. Introduction

Pediatric obesity is a key health concern worldwide. A recent systematic review and meta-analysis highlighted how children living in the Mediterranean countries have the highest prevalence rates of overweight/obesity (24% to 37%) in Europe [1,2]. In addition to the presence of cardiometabolic risk factors [3], children with obesity are more likely to experience physical and psychosocial distress (musculoskeletal pain, acute injuries, poorer balance and coordination, weight stigma, victimization) [4], which limits physical activity. Moreover, in children and adolescents with obesity, low levels of physical activity are consistently associated with a worse performance in several components of health-related physical fitness, such as cardiorespiratory fitness and muscle strength [4], leading to a vicious circle that worsens the severity of obesity and negatively impacts health-related quality of life (HRQOL) [5,6,7].

Evidence of lower physical functioning, health-related physical fitness and physical activity levels in youth with obesity has been shown in treatment-seeking samples, while few data are available in community samples. It has been questioned whether the impairment in physical and psychological health found in treatment samples may be also conveyed to non-treatment-seeking individuals. As far as we know, only one study performed on a clinical sample of youth with obesity showed lower scores in total, physical and psychosocial domains of quality of life compared to a community sample [8].

Since it is hard for obese youth to engage in physical activity, it is necessary to design physical activity interventions tailored to their abilities in order to favor adherence. Several studies have shown that school-based multicomponent interventions were effective in improving levels of physical activity and eating behavior in children and adolescents with overweight and obesity [9,10,11].

We speculated that a clinical sample of youth with obesity might suffer from psychosocial or health complaints leading to higher perceived difficulties in physical tasks and lower physical fitness than their non-treatment-seeking counterpart, such as students. If our hypothesis might be confirmed, the contribution of school-based programs of physical activity might be useful to supply current obesity management [12].

Against this background, our aim was to assess whether perceived difficulties in physical tasks and performance in health-related physical fitness differed between treatment- and non-treatment-seeking youths with obesity, respectively enrolled from a clinical and a school setting.

## 2. Materials and Methods

This cross-sectional study was performed from 1 April 2017 to 31 December 2017. In this period, 288 children and adolescents with overweight or obesity (54.9% males, mean age 10.8 years, range 7–16 years) were consecutively admitted for a first visit to the Obesity Unity of the Santobono Pausilipon Children Hospital of Naples, Italy; 308 children and adolescents were recruited from two local schools (one primary and one middle school) in the same period. For inclusion in the study, the following criteria were considered: age 8–14 years; BMI ≥ 97th percentile [13]. The exclusion criteria were cognitive or physical limitations in undergoing physical fitness tests. Finally, two hundred sixty-nine children and adolescents (55.0% males, mean age 10.5, range age 8–14 years) from the clinic and eighty-two (26.6% of the total sample) children and adolescents (61.0% males, mean age 10.3, range age 8–14 years) from the schools met the inclusion criteria and were effectively enrolled in this cross-sectional study. All children and their parents or legal guardians agreed to sign the written informed consent for all procedures before the enrolment. This study was approved by the Institutional Review Board of the Ospedale Cardarelli, Naples (reference number 33/28.03.2017), for children/adolescents recruited in the clinic and by the Institutional Review Board of Federico II University of Naples (reference number: 42/17. 21.03.2017) for children/adolescents recruited in school.

### 2.1. Anthropometric Measurements

The participants were measured by a skilled operator following standard procedures. Stature and body weight were measured with the participants wearing only light clothes and no shoes. Body weight and height were measured to the nearest 0.1 kg and 0.5 cm, respectively, using a medical scale and a wall-mounted stadiometer. BMI was calculated as weight/stature^2^ (kg/m^2^) and transformed into BMI Z-scores according to the WHO growth reference for school-age children and adolescents [13].

### 2.2. Questionnaires: Sports Participation and Sedentary Habits

The study included a questionnaire assessment by interview regarding sports participation in the previous 6 months (yes/no) and sedentary habits, as the sum of the daily hours spent watching television, playing video games, and surfing on the computer.

### 2.3. Questionnaires: Perceived Difficulties

Perceived difficulties with physical tasks were assessed by interview using a structured questionnaire, which included four questions regarding physical difficulties related to daily movement (walking, running, jumping and climbing stairs) [14]. Four possible answers (0 = never; 1 = sometimes; 2 = often; 3 = always) were available for each item. Perceived difficulty was defined when the perception was rated as ≥1 in each item.

### 2.4. Physical Fitness Assessment

Selected physical tasks performed in daily life or in sports activities were assessed according to standard procedures:

(1) Six-minute walking test was performed indoors [15]. The walking course length was 20 m along a corridor marked every 2 m with brightly colored tape. Cones were placed at either end of the walking course to indicate the beginning and the end points. Each participant was tested individually. Participants were informed that the purpose of the test was to assess how far they could walk in 6 min and were instructed to walk the longest distance possible during the allowed time. Hopping, skipping, running and jumping were not allowed during the test. Only the standardized phrases for encouragement (e.g., “keep going”, “you are doing well”) and announcement of time remaining were given. The six-minute walking distance (SMWD) was expressed in meters.
(2) Long jump (LJ) was used to assess lower body muscle strength [16]. Participants performed a two-foot take-off and landing. The swinging of the arms and flexing of the knees were permitted to provide forward drive. Participants attempted to jump as far as possible, landing on both feet without falling backward. Length was measured to the nearest point of contact on the landing. Two attempts were performed and the best value was used for analysis. Results were expressed in centimeters.

### 2.5. Statistical Analysis

Results are reported as mean ± standard deviation or percentage. Statistical significance was pre-determined as *p* < 0.05. All statistical analyses were performed using the Statistical Package for Social Sciences (SPSS Inc, Chicago, IL, USA) version 26. The Shapiro–Wilks normality test was applied to assess the normality of data distribution. Non-parametric variables were logarithmically transformed, but they were expressed as untransformed values for clarity of interpretation. Differences among groups were assessed using one-way ANOVA or a general linear model (when data were adjusted for age). The relationship between categorical variables (among BMI Z-score groups) was assessed by using the chi-squared linear-by-linear association (Mantel–Haenzsel test for trend). The Spearman’s rank correlation test was applied to assess the association between the results of fitness tests and perception of impairment in running, walking, jumping and stair climbing. The logistic regression analysis was used to estimate the relationship between perceived difficulties and other variables (setting, sex, age, BMI Z-score, sports participation and total sedentary time). The general linear model was used to assess the association between physical fitness tests and settings including possible confounders (sex, age, BMI Z-score, sports participation and total sedentary time).

## 3. Results

General characteristics, lifestyle behaviors and physical fitness of the study sample, stratified according to the setting, are shown in Table 1. A slightly lower prevalence of boys (albeit not significant) and significantly higher values of BMI Z-score (*p* < 0.001) were found in the hospital setting compared to the school setting. Youths from the hospital were more sedentary and performed worse in SMWD and LJ. Higher perceived difficulties in walking, running, jumping and climbing stairs were reported in youths from the hospital compared to the school (Table 1).

Correlation analyses between the two physical fitness tests and perceived difficulties in each task were performed in the total sample and in the two study groups. In the total sample, a moderate association was found between SMWD and perceived difficulty in walking or running (r = −0.378 and r = −0.407, respectively), while only a slight (but significant) association was found between LJ and perceived difficulty in jumping and stair climbing (r = −0.238 and r = −0.207, respectively) (Supplementary Table S1). Considering the two study groups separately, only in the hospital group were slight negative correlations found between SMWD and perceived difficulty in walking or running (r = −0.193 and r = −0.206, respectively) and between LJ distance and perceived difficulty in jumping or stair climbing (r = −0.187, *p* and r = −0.149, respectively) (Supplementary Tables S2 and S3).

**Figure 1:**
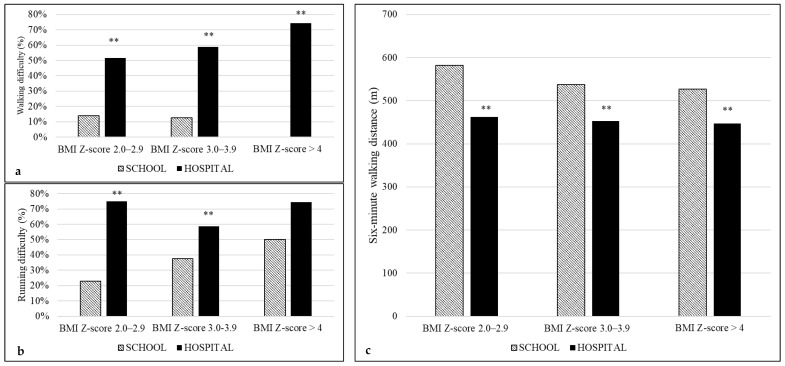
Perceived difficulties and performance in the two settings stratified by BMI Z-score categories: (**a**) walking difficulty (chi-squared linear-by-linear association for school *p* = 0.332; for hospital *p* = 0.011); (**b**) running difficulty (chi-squared linear-by-linear association for school *p* = 0.069; for hospital *p* = 0.357); (**c**) six-minute walking distance performance (ANOVA overall for school *p* = 0.017; for hospital *p* = 0.139). ** *p* < 0.001 hospital vs. school.

Perceived difficulties in walking and running and the respective performance in SMWD according to categories of BMI Z-score are shown in Figure 1. For the same BMI Z-score category, the perceived difficulties as well as walking and jumping performances were lower in the hospital group compared to the school group.

**Figure 2:**
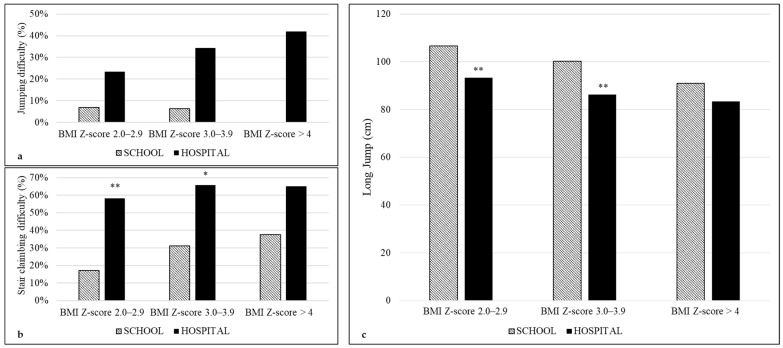
Perceived difficulties and performance in the two settings stratified by BMI Z-score categories: (**a**) jumping difficulty (chi-squared linear-by-linear association for school *p* = 0.507; for hospital *p* = 0.013); (**b**) stair climbing difficulty (chi-squared linear-by-linear association for school *p* = 0.109; for hospital *p* = 0.279); (**c**) long jump performance (ANOVA overall for school *p* = 0.081; for hospital *p* = 0.002). * *p* < 0.05 hospital vs. school; ** *p* < 0.001 hospital vs. school.

A significant trend across the BMI Z-score categories was found for the perceived difficulties in walking and jumping only in the hospital group (*p* = 0.011 and *p* = 0.013, respectively) (Figure 1 and Figure 2). In addition, considering the physical fitness tests, a significant trend among BMI categories was found for SMWD in the school group (*p* = 0.017) and for LJ in the hospital group (*p* = 0.002) (Figure 1 and Figure 2).

Logistic regression analysis was run to analyze the variables associated with the perceived difficulties, such as sex, age, BMI Z-score, sports participation and total sedentary time; results are shown in Table 2 (only the significant predictors are shown).

The hospital setting was a predictor of all perceived difficulties (*p* < 0.05). In particular, compared to the school setting, youths from the hospital setting were more likely to report physical difficulties in daily tasks (OR 4.6 for stair climbing and 7.8 for walking), independently of the other variables. Perceived difficulties in tasks related to vigorous efforts, such as running and jumping (OR 1.6 for both), were also significantly associated with BMI Z-score, while walking (OR 1.2) and running (OR 1.3) were also associated with sedentary time.

The association between physical fitness tests and settings was evaluated by a general linear model that included possible confounders such as age, sex, BMI Z-score, sports participation and total sedentary time; results are depicted in Table 3 (only significant variables are shown). The hospital setting was a negative predictor (*p* < 0.05), while age was a positive predictor of both SMWD and LJ: setting, sex, age and sedentary habits explained 50% of the total variance for SMWD.

## 4. Discussion

The main results of this study showed that youths with obesity attending a hospital setting had higher perceived difficulties in physical tasks and lower lifestyle habits and performed worse in physical fitness tests compared to non-treatment-seeking youths recruited in a school setting. Indeed, youths from a hospital setting were more physically impaired than youths from a school setting. The hospital setting was an independent predictor of both perceived difficulties and physical performance, after controlling for age, sex, BMI Z-score and lifestyle habits.

Pediatric obesity may be considered as a condition of disability, as far as functional mobility and participation in the activities of daily living are involved [4]. Therefore, counseling about changes in lifestyle cannot disregard the assessment of perceived difficulties in daily physical tasks and real functional mobility. To date, a huge body of literature about the negative consequences of obesity on the physical and psychological aspects of quality of life, lifestyle habits and/or physical fitness has been produced in treatment-seeking youths with obesity compared to normal-weight individuals [17,18]. Similarly, children with obesity enrolled from a community sample had lower physical and social functioning scores compared with non-overweight children [8,19,20]. Less explored is the comparison between treatment- and non-treatment-seeking samples. This issue is not of secondary importance in modeling treatment or preventive strategies based on the promotion of physical activity in different settings [21]. In particular, to the best of our knowledge, no previous study has compared perceived difficulties in physical tasks and performance in physical fitness tests between youths with obesity from a clinical setting and a school setting. Therefore, we assessed difficulties in those tasks that are routinely performed by children (such as walking, running, jumping and stair climbing) and that provide opportunities to engage in physical activities at home, at school and in the community and contribute to health-related quality of life. Indeed, items exploring physical mobility are included in questionnaires on the health-related quality of life in children and adolescents [22]. In addition, we objectively assessed the corresponding tasks, such as walking and jumping, that are considered markers of cardiorespiratory endurance and lower body strength, respectively. We found that performance in SMWD significantly decreased with the increasing severity of obesity only in the school sample (Figure 1); on the contrary, LJ decreased with the increasing severity of obesity, only in the hospital sample (Figure 2). However, for the same BMI Z-score category, there was a larger discrepancy in the perceived difficulties compared to that found in the physical performance between hospital and school groups (Figure 1 and Figure 2). Logistic regression analysis and general linear modeling confirmed that the hospital setting was a negative predictor of both perception of functioning (mainly for walking) and physical performance (mainly for the SMWD). Our findings are supported by a previous study, which demonstrated better scores in total, physical and psychosocial domains of quality of life in a community sample of obese children compared to a severely obese clinical sample [8]. Interestingly, differences in physical and psychosocial quality of life were found between inpatient and outpatient youths with obesity [23].

The evaluation based on performance might be incomplete without assessing the subjective perception in doing that performance. Slight to moderate correlations between perceived difficulties and the corresponding tasks were found in the whole sample but were confirmed only in the hospital setting by separate analyses. It has been reported that the perception of motor competence may mediate the relationship between the real motor competence and physical activity [24]. In fact, physiological and emotional responses associated with task difficulties may be important determinants of self-efficacy [25]. A negative association between perceptions of task difficulties and self-efficacy may influence any attempt to engage in physical activity [26].

The two groups showed significant differences in sedentary time, but not in sports participation. Perceived difficulties in walking and running and the SMWD were independently associated with sedentary time. The lack of correlation with physical activity, which has been suggested by others, can be probably explained by the fact that we analyzed only sports participation, which may have cost and time barriers compared to overall physical activity. Conversely, jumping performance was positively influenced by sports participation, suggesting the effect of training on muscle strength. Previous studies described a lower performance in SMWD and LJ in youths with obesity compared to normal-weight samples [18,27,28,29] and an association between components of physical fitness and lifestyle habits (such as physical activity and screen time) [30]. In our sample, BMI Z-score was independently associated with perceived difficulty in tasks related to a more vigorous effort (running and jumping) and with the LJ performance, a test that requires higher body displacement and limb strength than walking. Indeed, the higher mechanical overload in youths with obesity overburdens the muscles, explaining the negative association with performance [31,32].

Our study presents some limitations: it has a cross-sectional design; hence, we cannot assess the direction of the association between BMI SDS, perceived difficulties and physical fitness tests. This study presents also several strengths: the anthropometric data and the physical fitness tests were assessed by the same researchers in both settings; further, the physical fitness tests selected in the domain of cardiorespiratory fitness and strength are supported by a wide body of literature for their features of high feasibility and reproducibility [16].

## 5. Conclusions

By pointing out differences between treatment- and non-treatment-seeking youths, this study extends the research findings on the physical and psychological consequences of pediatric obesity. Youths attending a clinical setting most reasonably suffer from social or health complaints, with a gradient based on the severity of obesity. In fact, treatment-seeking youths are more distressed in terms of BMI Z-scores, perceived difficulties and fitness performance compared to non-treatment-seeking youths with obesity. These differences are only in part explained by obesity severity, since they persisted in adjusted comparisons. In perspective, pediatricians, kinesiologists and other healthcare professionals should consider the physical aspects imposed by obesity and the physiological and emotional consequences on self-efficacy in order to treat this condition with appropriate and personalized modalities. Lastly, considering the central role of schools as inclusive communities, the results of our study support the implementation of programs promoting physical activity and fitness for obesity prevention and treatment in the school setting.

## Figures and Tables

**Table 1:** General characteristics, lifestyle behaviors and physical fitness in the study samples, stratified according to setting.

	School (n = 82)	Hospital (n = 269)	*p*
Boys N (%)	50 (61.0)	148 (55.0)	
Age (years)	10.4	10.6	0.317
Weight (kg)	56.3	63.2	<0.001
Stature (cm)	146.0	146.0	0.981
BMI Z-score	2.8	3.3	<0.001
Sports participation N (%)	37 (45)	(121) 45	0.982
Sedentary time (h/day)	3.2	4.5	<0.001
Six-minute walking distance (m)	568	456	<0.001
Long jump distance (cm)	103.4	89.1	<0.001
Perceived difficulties			
Walking N (%)	10 (12)	156 (58)	<0.001
Running N (%)	23 (28)	210 (78)	<0.001
Jumping N (%)	5 (6)	81 (30)	<0.001
Stair climbing N (%)	18 (22)	167 (62)	<0.001
BMI Z-score categories			
2.0–2.9 N (%)	58 (70.7)	124 (46.1)	<0.001
3.0–3.9 N (%)	16 (19.5)	102 (37.9)	
4.0–4.9 N (%)	8 (9.8)	43 (16.0)	

BMI = body mass index; data are expressed as mean ± standard deviation or number (percentages) as appropriate.

**Table 2:** Odds ratio associating perceived difficulties with specific demographic and other variables.

Perceived Difficulty	Variables (*p* in Brackets)	OR	95% C.I.	
Walking	Setting * (<0.001)	7.846	3.779	16.289
	Sedentary time * (0.010)	1.171	1.039	1.320
Running	Setting * (<0.001)	6.197	3.400	11.292
	BMI Z-score * (0.015)	1.633	1.100	2.427
	Sedentary time * (0.001)	1.274	1.100	1.475
	Sex * (0.049)	1.763	1.023	3.143
Jumping	Setting * (<0.001)	5.688	2.158	14.996
	BMI Z-score * (0.006)	1.578	1.133	2.196
Stair Climbing	Setting * (<0.001)	4.627	2.523	8.486

* = *p* < 0.05 for each coefficient; abbreviations: OR = odds ratio; C.I. = confidence interval; BMI = body mass index.

**Table 3:** Significant predictors of health-related physical fitness tests.

Significant Predictors (Standardized Coefficients in Brackets)		R^2^	SEE
Six-minute walking distance	Setting * (−0.642), age * (0.208), sedentary habits * (−0.132), sex * (0.088)	0.496	50.207
Long jump distance	Setting * (−0.252), sex * (0.279), BMI Z-score * (−0.200), age * (0.137), sports participation * (0.117)	0.243	17.776

* = *p* < 0.05 for each coefficient; abbreviation: SEE = standard error of estimate.

## Data Availability

Not applicable.

## Supplementary Materials

The following supporting information can be downloaded at: https://www.mdpi.com/article/10.3390/children9091351/s1, Table S1: Correlation between physical fitness tests and perception of impairment in running, walking, jumping and stairs climbing in the total sample; Table S2: Correlation between physical fitness tests and perception of impairment in running, walking, jumping and stairs climbing in youths from hospital; Table S3: Correlation between physical fitness tests and perception of impairment in running, walking, jumping and stairs climbing in youths from school.
