# Supplementary material for: Perceived Difficulties in Physical Tasks and Physical Fitness in Treatment- and Non-Treatment-Seeking Youths with Obesity
Source: Children (Basel). 2022 Sep 4;9(9):1351. doi: 10.3390/children9091351 (PMC9498025; doi:10.3390/children9091351)
Supplement: Supplementary file 1 [file children-09-01351-s001.zip › children-1865953-supplementary.pdf]

Supplementary Table S1. Correlation between physical fitness tests and perception of impairment in running, walking, jumping and stairs climbing in the total sample.

	WALKING	RUNNING	STAIRS CLIMBING	JUMPING
Six-minute walking distance	-0.378	-0.407	-0.233	-0.173
P	<0.001	<0.001	<0.001	<0.001
Long Jump	-0.158	-0.218	-0.207	-0.238
P	0.003	<0.001	<0.001	<0.001

Supplementary Table S2. Correlation between physical fitness tests and perception of impairment in running, walking, jumping and stairs climbing in youths from hospital.

	WALKING	RUNNING	STAIRS CLIMBING	JUMPING
Six-minute walking distance	-0.206	-0.193	-0.045	-0.026
P	0.001	0.001	0.458	0.677
Long Jump	-0.037	-0.079	-0.149	-0.187
P	0.542	0.197	0.015	0.002

Supplementary Table S3. Correlation between physical fitness tests and perception of impairment in running, walking, jumping and stairs climbing in youths from school.

	WALKING	RUNNING	STAIRS CLIMBING	JUMPING
Six-minute walking distance	-0.039	-0.130	-0.044	-0.162
p	0.731	0.247	0.697	0.147
Long Jump	-0.111	-0.180	0.017	-0.129
p	0.320	0.107	0.881	0.247
